# Impact of COVID-19 on orthopaedic residents—an Indian perspective

**DOI:** 10.1097/OI9.0000000000000096

**Published:** 2021-03-15

**Authors:** Prasanna Kumar GS, Amit Kumar Yadav, Abhishek Harsoor, Kantharaju H, Akash V Mane, Nikhil D. Palange

**Affiliations:** a Department of Orthopaedics, Grant Govt Medical College and Sir Jj Group of Hospitals, Mumbai; b Department of Orthopaedics, Govt Medical College, Nagpur; c Department of Orthopaedics, Grant Govt Medical College, Mumbai, India.

**Keywords:** COVID-19, impact, orthopaedics, residents

## Abstract

**Objectives:**

The COVID-19 pandemic is a public health emergency causing a deleterious effect on the health system. It affected all the specialties and subspecialties in the medical field causing havoc in the health institutions. This pandemic affected both orthopaedic consultants and the residents who are under training. Our purpose was to study the impact of COVID-19 on orthopaedic residents in their professional life.

**Method:**

The study design was a computer-based digital online survey of the orthopaedic residents in India. The survey had 15 questions with multiple options related to the effect of COVID-19 on their orthopedic department, effect on teaching, surgical exposure, hands-on surgeries, the effect on workload, effect on mental stress, exposure to arthroplasty, arthroscopic surgeries, spine surgeries, and deformity correction surgeries.

**Results:**

Elective surgeries stopped in 91% of the hospitals, academic teaching stopped in 98% of the institutions. Eighty-six percent of the residents are not getting adequate surgical exposure, 73% of the residents are getting negligible hands-on surgical training. Residents are mentally stressed related to academic examinations, academic training, and also because of COVID 19 duties. Residents are getting the least exposure in subspecialties like arthroplasty, arthroscopy, and spine.

**Conclusion:**

The COVID-19 pandemic not only affected the orthopaedic consultants but also the orthopaedic residents to a great extent as residents are the backbone of any department/institution. The pandemic affected significantly resident's academic teaching, surgical exposure, hands-on training and mental stress related to COVID duties, academic training disturbance, and also academic examinations.

## 1 Introduction

In December 2019, a new type of pneumonia was recognized in Wuhan (China), and was identified by the WHO as a novel strain of coronavirus (2019-nCoV).^[1–3]^ The infection has continued to spread across the globe and was declared a public health emergency of international concern on January 30, 2020 and later as a pandemic on March 11, 2020 by the WHO.^[4]^ On February 11, 2020, WHO declared this as COVID-19 (CORONA VIRUS DISEASE-19).^[5]^

Health care workers working as Frontline warriors have been recognized as high-risk groups since the beginning of the outbreak.^[6]^ Physicians, anesthetists, pulmonologists, and emergency medicine doctors are at the front line in treating COVID patients and are at high risk of getting severely infected while treating these patients. Orthopaedic surgeons are also affected by this crisis although they are not the frontline workers and are working outside their field of specialization to combat the COVID crisis. Operating theaters are converted into intensive care units to treat these patients. Orthopaedic surgeries in this pandemic are reserved only for trauma, limb-saving injuries, cancers, and severe infections.^[7]^

Orthopaedic residents are also affected by this COVID 19 pandemic crisis as they are not getting enough surgical exposure and hands-on training as routine surgeries are canceled or postponed at this time. Orthopaedic residents are also working with physicians in corona wards to fight against COVID along with their routine orthopaedic duties. Most of the subspecialty surgeries in orthopaedics like joint replacement surgeries, arthroscopy, and sports injury surgeries, elective spine, and deformity correction surgeries are postponed or canceled as these are not emergencies. So the exposure in these above-mentioned fields is negligible to the orthopaedic residents under training at present.

The purpose of this study was to analyze the impact of the COVID-19 crisis on orthopaedic residents under training in their professional life and the tactic used was a carefully constructed survey.

## 2 Materials and methods

**Figure 1:**
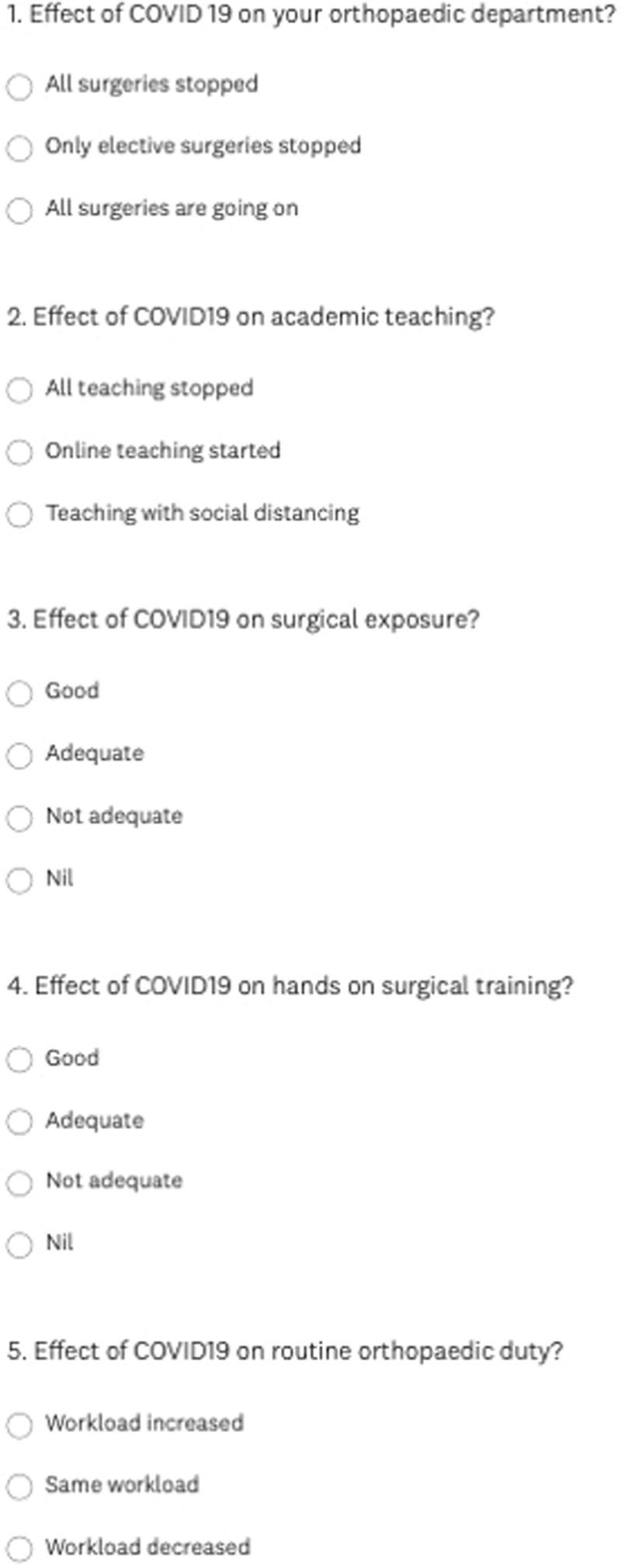
Survey questionnaire (1–5).

**Figure 2:**
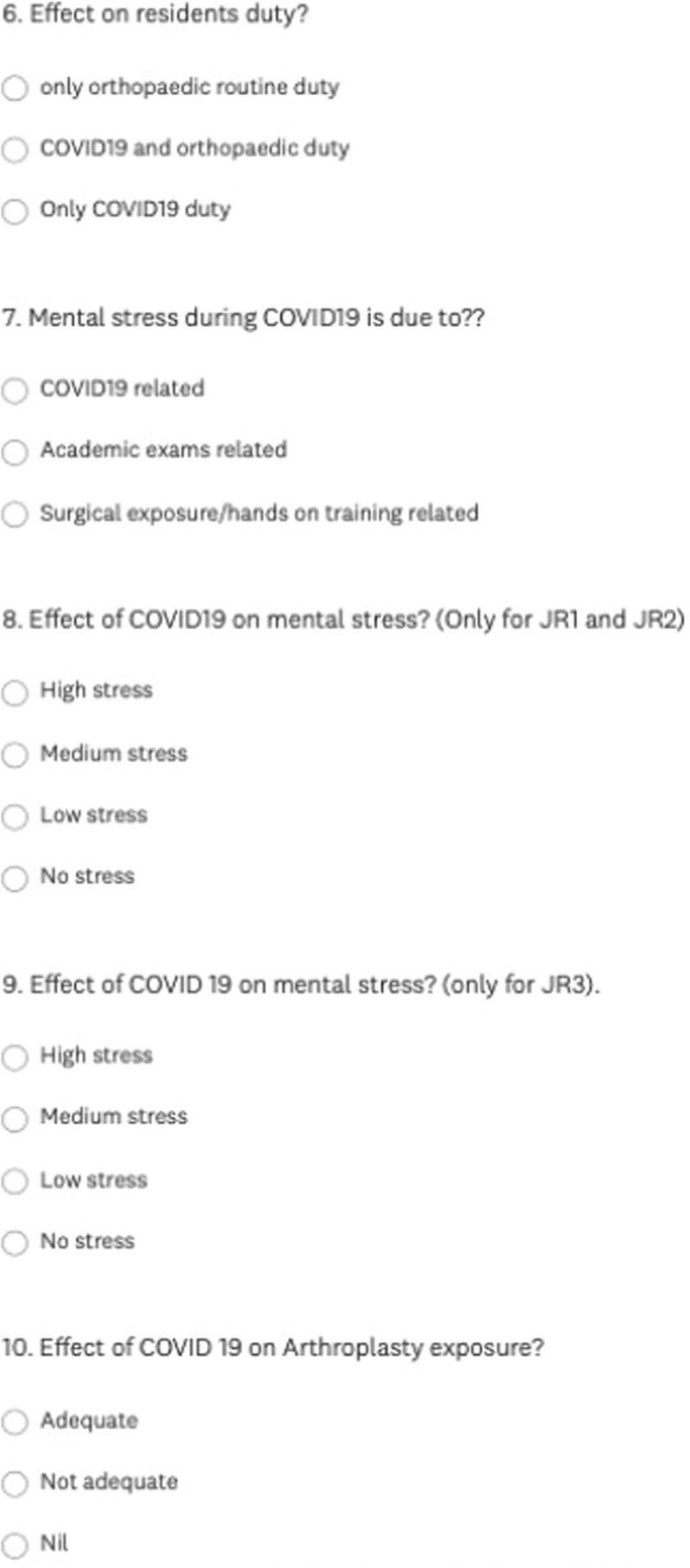
Survey questionnaire (6–10).

**Figure 3:**
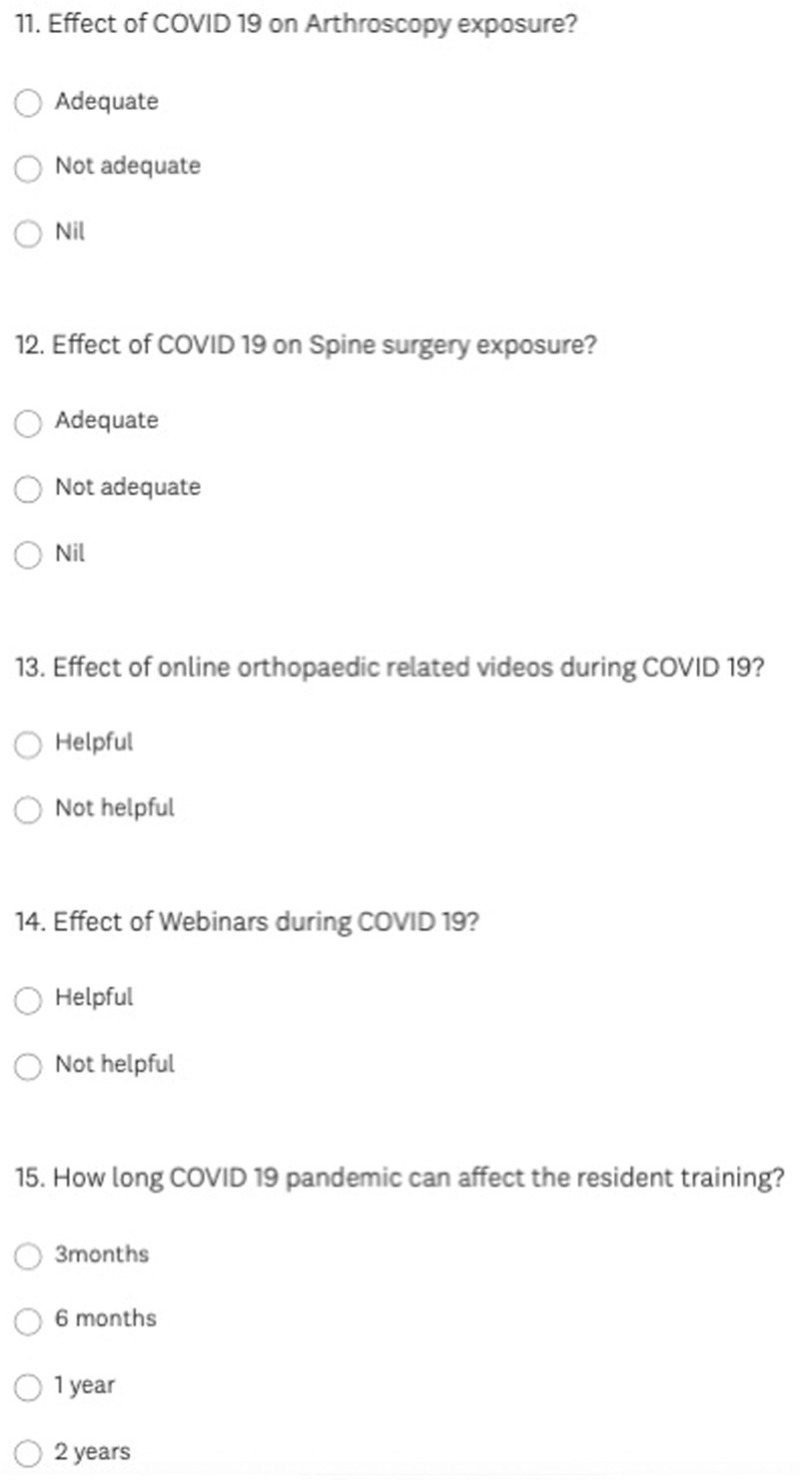
Survey questionnaire (11–15).

It was a computer-based digital online survey of the orthopaedic residents in India. The survey had 15 questions with multiple options related to the effect of COVID-19 on their orthopedic department, effect on teaching, surgical exposure, hands-on surgeries, the effect on workload, effect on mental stress, exposure to arthroplasty, arthroscopic surgeries, spine surgeries, and deformity correction surgeries (Figs. 1–3).

We also surveyed the effect of online orthopaedic videos, the effect of webinars on academics, and also about the level of personal protection during COVID in orthopaedic residents.

Only junior residents (JR) were included in the study. JR are the candidates who joined the orthopaedic training after the MBBS training in India. They have to work for 3 years as junior residents (as JR1 in the first year, JR2 in the second year, and JR3 in the third year) in the orthopedic department and have to pass an academic examination at the end of third year to get an orthopaedic postgraduation degree. After this degree, they are allowed to practice independently as orthopaedic surgeons. Senior residents are the candidates, who have passed their MS orthopaedic examination and completed their orthopaedic training. Senior residents will not come under orthopaedic trainees, so we did not include them in the study.

The above questionnaire (multiple choice questions) was sent to multiple residents groups through social media like what's app, telegram, and through email, on April 6, 2020, and the residents’ replies were collected for 1 week (till April 12), which had 1214 residents reply and results were tabulated using standard statistical techniques on an Excel Spreadsheet. The questionnaire was sent only one time for the residents and the response rate was fairly representative of all of India.

## 3 Results

India is facing the effects of a pandemic like any other country in the world. As India is more populated, and a developing country, the effects of the pandemic are more. India was in complete lockdown from March 2020 to May 2020. At present most of the states in the country are still following the norms of lockdown in containment areas.

Out of 1214 residents, 836 (69%) replied to the survey and in that group 18% were junior resident 1 (JR 1), 35% were junior resident 2 (JR2), and 47% were junior resident 3 (JR3). Only elective surgeries have stopped in 91% of the hospitals, in 7% of the hospitals all the surgeries including emergencies have stopped, and in 2% of the hospitals all surgeries are going on.

**Figure 4:**
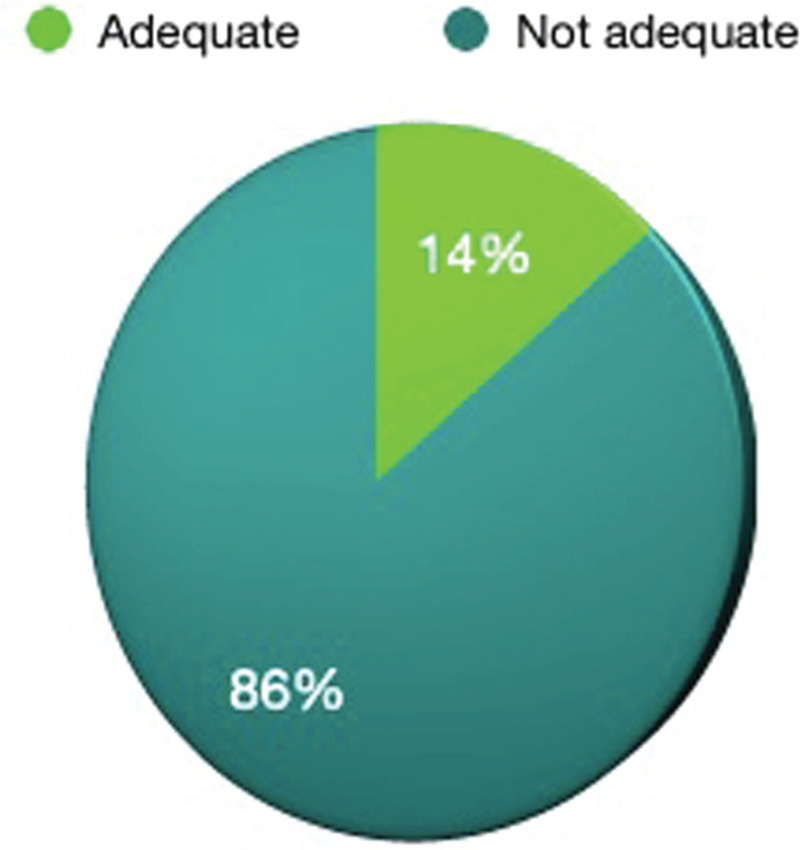
Showing effect of COVID 19 on residents’ surgical exposure.

**Figure 5:**
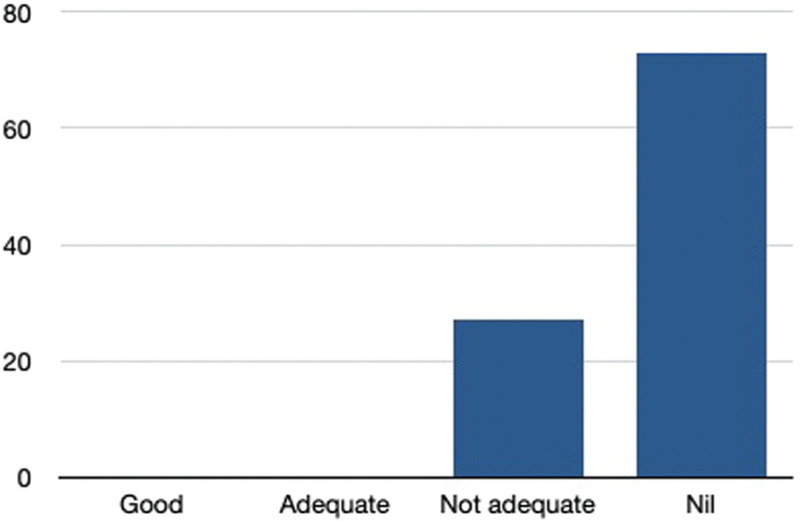
Showing effects of COVID 19 on residents’ hands on surgical training.

With regards to residents’ teaching, all the academic teaching has stopped in 98% of the institutions, there was online teaching in 2% and none of the colleges/hospitals are doing teaching with social distancing. Concerning the surgical exposure, 86% of the residents are not getting adequate exposure, only 14% are getting adequate surgical exposure and none are getting good exposure during this COVID time (Fig. 4). Seventy-three percent of the residents are not at all getting hands-on surgeries, 27% are getting inadequate surgeries, and none of the residents are getting adequate and good surgical hands-on exposure (Fig. 5). According to 96% of the residents, the orthopaedic routine workload has decreased and in 4% of the residents, the workload is the same as before COVID time. Fifty-three percent of the residents are doing both COVID and orthopaedic routine duties, 30% of residents are doing only orthopaedic duties, and 17% of the residents are doing exclusively COVID duties.

COVID 19 has highly affected 91% and moderately affected 9% of the examination going junior residents 3 (JR3) due to mental stress because of the postponement in academic examinations along with COVID duties. Forty percent of the residents are facing mental stress related to examinations (in this all are junior resident 3), 30% have mental stress related to surgical exposure/hands-on surgeries/academic teaching (mainly junior residents 2), and 30% are facing COVID-related mental stress.

With regards to exposure in subspecialties of orthopaedics like arthroplasty, arthroscopy, spine and deformity correction, 99% of the residents are not at all getting any exposure in arthroplasty and arthroscopy whereas in spine 89% of residents are not getting exposure and only 11% are getting exposure.

The effect of online videos and webinars on the residents during COVID times for academic purposes showed that 40% of the residents are getting helped by online videos and webinars to learn and improve their academic knowledge related to orthopaedics (Table 1).

**Table 1:** Showing effects of COVID 19 on orthopaedic residents.

Effects of COVID 19		Percentage (%)
Effects on department	All surgeries stopped	7
	Only elective surgeries stopped	91
	All surgeries going on	2
Effect on academic teaching	All teaching stopped	98
	Online teaching	2
	Teaching with social distancing	Nil
Effect on surgical exposure	Good	Nil
	Adequate	14
	Not adequate	86
Effect on hands on surgical training	Good	Nil
	Adequate	Nil
	Not adequate	27
	Nil	73
Effect on routine orthopaedic duty	Workload decreased	96
	Same workload	4
	Workload increased	Nil
Effect on residents duty	Only orthopaedic routine	30
	Covid and orthopaedic duty	53
	Only Covid duty	17
Effect on arthroplasty and arthroscopy exposure	Adequate	Nil
	Not adequate	1
	Nil	99
Effect on spine exposure	Adequate	Nil
	Not adequate	11
	Nil	89
Use of webinars and online videos as academic tool	Helpful	40
	Not helpful	60

According to the survey by the residents, the time up to which COVID 19 would be there affecting clinical and surgical practice, 11% of residents suggested 3 months, 80% suggested 6 months to 1 year, and 9% suggested more than 1 year.

## 4 Discussion

The COVID-19 pandemic is not only affecting the medical fields but also has a great influential impact on surgical fields like orthopaedics, as routine outpatient care and elective surgeries are getting hampered. At present all the routine orthopaedic surgeries are postponed /canceled because of this pandemic. According to our survey, 91% of the hospitals have stopped doing elective surgeries and 7% of the hospitals have stopped all surgeries including emergencies because these hospitals are converted into exclusive COVID hospitals to take care of only patients affected by coronavirus. In these exclusive COVID hospitals, all the specialty, and super specialty doctors are treating pandemic patients leaving the specialty in which they are trained. According to Graichen,^[8]^ the quality of orthopaedics work at present during the pandemic depends on his or her general medical training and experience, and the role is assisting rather than leading.

In 98% of the institutions, the academic teaching like seminars, workshops, case presentations, journal club, and ward rounds discussion has stopped during the pandemic to prevent overcrowding and to maintain social distancing as many are asymptomatic carriers. Some of the institutions have started an online teaching program for residents like online lectures and interactive-based discussions.^[9]^ To prevent the loss of academic teaching to the residents in this pandemic time, the use of the online platform is the way forward. While didactic lectures are still very important in the medical education system, but newer initiatives like webcasts are more utilized nowadays and E-learning platforms and ^[10]^ videoconferencing^[11]^ can be used to demonstrate medical procedures and surgical techniques. Generally, there was about 30 to 40 hours of postgraduate training per week before the COVID 19 pandemic and it is reduced to around 6 to 10 hours of training per week in some hospitals with no teaching in most of the centers as we are still in the midst of the pandemic.

In India, since we are still in the midst of the pandemic and cases are increasing day by day, the effect of COVID 19 on academic teaching is the same. The effects will be long term as the pandemic is still high in India.

Surgical exposure and hands-on surgery training have drastically come down because of the COVID 19 pandemic, 86% of the residents are not getting adequate surgical exposure and 73% of the residents are not at all getting any hands-on training. This is because of the cancellation of all elective surgeries in orthopaedics which are usually done by residents. As well, emergency cases are performed by senior doctors nowadays to prevent excessive time wastage during the surgery and to finish the surgery early so that exposure time in the operation theater is less to the operating surgeon as well as to the anesthetists and also to reduce the number of residents getting washed up for assisting to prevent overcrowding. According to 96% of the residents, the orthopaedic routine workload has decreased, because of the cancellation of all routine surgeries and also fewer emergency and trauma cases as there are fewer road traffic accidents during this pandemic and lockdown period. The number of surgeries performed or assisted by orthopaedic residents during the pandemic is less compared to the residents who have completed their training in 2019 or before the pandemic.

As 53% of orthopaedic residents are doing both orthopaedic routine and COVID duties and 17% of the residents are doing exclusive COVID duties, it is essential to give proper guidance about the work and the personal protection, to prevent the residents from getting infected by COVID 19 and to give proper quarantine and rest to improve the efficiency of the residents. In India, some medical colleges and hospitals are converted into exclusive COVID 19 hospitals, in which only patients with COVID are taken care of. The residents who were there before this pandemic in the above-mentioned exclusive COVID 19 hospitals are working in the same hospitals by treating only COVID patients. This 17% of the residents are not at all getting any exposure related to orthopaedic training apart from COVID 19.

Residents are facing mental stress during this pandemic related to examinations, surgical exposure, and hands-on training and related to COVID duty. Ninety-one percent of the junior residents 3 (JR3) are facing high mental stress because their scheduled academic examinations are getting postponed/canceled and in some States, the examination dates have not yet been announced, along with these residents also doing COVID 19 duties. Most of the examination-going resident posts are getting extended and in some areas, this extended period is considered in their bond service. It is very important to give proper guidelines about the academic examinations to the residents and psychiatric advice if severely stressed to overcome these problems. Online examination conduction is one of the options that can be done during this pandemic period. Workload and coronavirus infection-related stress and psychological issues can be managed by giving information about, how to mentally prepare oneself in the frontline, the emotional challenges one would undergo, and tips for recognizing burnout, resources for support groups, helplines to call, and/or for direct consultation.^[12]^

The COVID 19 pandemic has strongly affected the subspecialties of orthopaedics like arthroplasty, arthroscopy, and spine, as most of these surgeries are elective. Residents are getting the least exposure in these subspecialties in their academic term in this pandemic. The residents who are in these subspecialty postings are having a huge loss in their academic year. The junior residents 2 (JR2) are at a great loss as their term is going to end in 6 months. The online videos showing the basics of these specialties and surgical steps can be made use of in this pandemic time to improve the knowledge about the subspecialties.

The effect of online videos and webinars conducted in this pandemic time showed that only 40% of the residents are getting benefit by the above because most of the webinars and online videos are based on case discussions, advanced techniques which are helpful for young orthopaedic surgeons. Webinars and online videos consist of basic orthopaedic sciences, case presentations, basic surgical steps, and procedures that help all the residents to improve their academic knowledge in the pandemic time. The Pandemic also affected the postgraduation counseling process of residents this academic year, because of this, the workload of junior residents 1(JR1) has increased and also their academic term has extended. The effect and impact on all the residents (JR 1, 2, and 3) will be more if the pandemic lasts for more than a number of months. The impact is more on JR3 if it lasts for 6 months to 1 year and JR1 and JR2 will be affected more if it lasts for more than 1 year in their academic training. Mental stress will be more if it lasts for more time as the residents lack in their academic training.

If the pandemic lasts for more time, the impact can be mitigated by online training of the residents by virtual surgical demonstration, clinical examination, case presentations, and discussions. There may be long term consequences, as the residency training is the crucial period to develop surgical skills and knowledge and if this is not proper, it can affect the future orthopaedic practice of the candidate.

## 5 Conclusion

COVID-19 not only affected the orthopaedic consultants but also the orthopaedic residents to a great extent as residents are the backbone of any department/institution. The Pandemic affected significantly residents academic teaching, surgical exposure, hands-on training, mental stress related to COVID duties, academic training disturbance, and also academic examinations. Academic teaching can be continued using online platforms showing basic essential orthopaedic procedures and techniques.
